# Consequences of Seed Origin and Biological Invasion for Early Establishment in Restoration of a North American Grass Species

**DOI:** 10.1371/journal.pone.0119889

**Published:** 2015-03-05

**Authors:** Mollie E. Herget, Kristina M. Hufford, Daniel L. Mummey, Lauren N. Shreading

**Affiliations:** 1 Department of Ecosystem Science and Management, University of Wyoming, Laramie, Wyoming, United States of America; 2 MPG Ranch, Florence, Montana, United States of America; University of Saskatchewan, CANADA

## Abstract

Local, wild-collected seeds of native plants are recommended for use in ecological restoration to maintain patterns of adaptive variation. However, some environments are so drastically altered by exotic, invasive weeds that original environmental conditions may no longer exist. Under these circumstances, cultivated varieties selected for improved germination and vigor may have a competitive advantage at highly disturbed sites. This study investigated differences in early establishment and seedling performance between wild and cultivated seed sources of the native grass, *Poa secunda*, both with and without competition from the invasive exotic grass, *Bromus tectorum*. We measured seedling survival and above-ground biomass at two experimental sites in western Montana, and found that the source of seeds selected for restoration can influence establishment at the restoration site. Cultivars had an overall advantage when compared with local genotypes, supporting evidence of greater vigor among cultivated varieties of native species. This advantage, however, declined rapidly in the presence of *B*. *tectorum* and most accessions were not significantly different for growth and survival in competition plots. Only one cultivar had a consistent advantage despite a strong decline in its performance when competing with invasive plants. As a result, cultivated varieties did not meet expectations for greater establishment and persistence relative to local genotypes in the presence of invasive, exotic species. We recommend the use of representative local or regional wild seed sources in restoration to minimize commercial selection, and a mix of individual accessions (wild, or cultivated when necessary) in highly invaded settings to capture vigorous genotypes and increase the odds native plants will establish at restoration sites.

## Introduction

Ecological restoration of native plants depends on successful germination and establishment of seeded species, and the source of seeds for restoration may have consequences for both short- and long-term population viability [1, 2]. Intraspecific variation is common within plant species that occupy a wide geographic range and span different environments [3], [4], and these diverse adaptive traits among populations may be critical for resilience to disturbance [5]. Species-level diversity may also drive community-level variation leading to broader ecological consequences [6–8]. As a result, guidelines for ecological restoration recommend the use of local seed sources to increase the likelihood that plants are adapted to site conditions, maintain genetic diversity and improve the long-term sustainability of restored plant populations [2, 9].

While local, wild-collected seed is recommended for ecological restoration, its availability is scarce and restoration practitioners frequently purchase commercially produced seed sources to meet native vegetation goals and reduce costs [10, 11]. Commercial seeds are often derived from cultivated plant varieties—or cultivars—and generally represent a limited number of populations of native species that are suitable for agricultural production. Cultivars are selected for improved germination, vigor and competitive ability, and are likely to represent low levels of genetic diversity and novel genotypes relative to remnant populations at restoration sites [12, 13]. Few data are available for the consequences of the use of cultivars in restoration, but recent evidence suggests that cultivated varieties differ from local genotypes in morphological and physiological traits, and may alter patterns of dominance within plant communities [8, 14, 15]. Large-scale introductions of novel genotypes may therefore have unintended consequences, such as the swamping of local genotypes, and altered community structure and function [1, 8].

Recommendations to use locally adapted seed sources are based on the assumption that the environmental conditions to which plants are adapted remain relatively unchanged. However, some areas are so drastically altered by exotic, invasive weeds that original environmental conditions may no longer exist [16, 17]. Because of their increased vigor, cultivars may have a competitive advantage at highly invaded restoration sites and the assumption that local genotypes are better adapted to site conditions than cultivars may no longer hold [18–20]. In light of widespread biological invasion, the geographic origin and genetic composition of seed stock may have significant consequences for restoration outcomes [1, 21]. Both the advantages and drawbacks of using cultivars and local genotypes for restoration require further investigation in the field.

The spread of *Bromus tectorum* L. (cheatgrass or downy brome) in North America is a well-documented example of land conversion as a result of biological invasion [22]. *Bromus tectorum* currently dominates millions of acres of grassland in the Intermountain West and represents a significant challenge for the restoration of native plant species [23]. *Bromus tectorum* is a winter annual and has a competitive advantage over native plants that germinate later during the growing season [24]. Stands of *B*. *tectorum* are also responsible for increased fire frequency, which can significantly alter both abiotic and biotic conditions at restoration sites [16]. Thus, the choice of appropriate seed sources for restoration may benefit from investigations of intraspecific variation and the performance of native plants in environments altered by biological invasion [25].

This study investigated the early seedling establishment and performance of wild and cultivated seed sources of *Poa secunda* J. Presl (Sandberg bluegrass) at sites impacted by *B*. *tectorum*. *Poa secunda* is a native, cool-season perennial grass commonly targeted for restoration because of its vast distribution [26] and potential ability to establish and compete for resources at sites invaded by exotic species [27]. We asked two questions relevant for grassland restoration: 1) Does the performance of wild seed sources differ significantly from cultivated seed sources? 2) Are cultivars more likely than their wild relatives to establish and persist in the presence of invasive, exotic weeds? If seed origin affects plant performance, the choice of seeds for reintroduction may represent an important consideration in restoration planning. At the same time, if evidence suggests cultivars are more likely to persist in the presence of *B*. *tectorum*, the selection of suitable seed sources may change with the level of invasion at the restoration site.

## Materials and Methods

### Study Area

**Fig 1:**
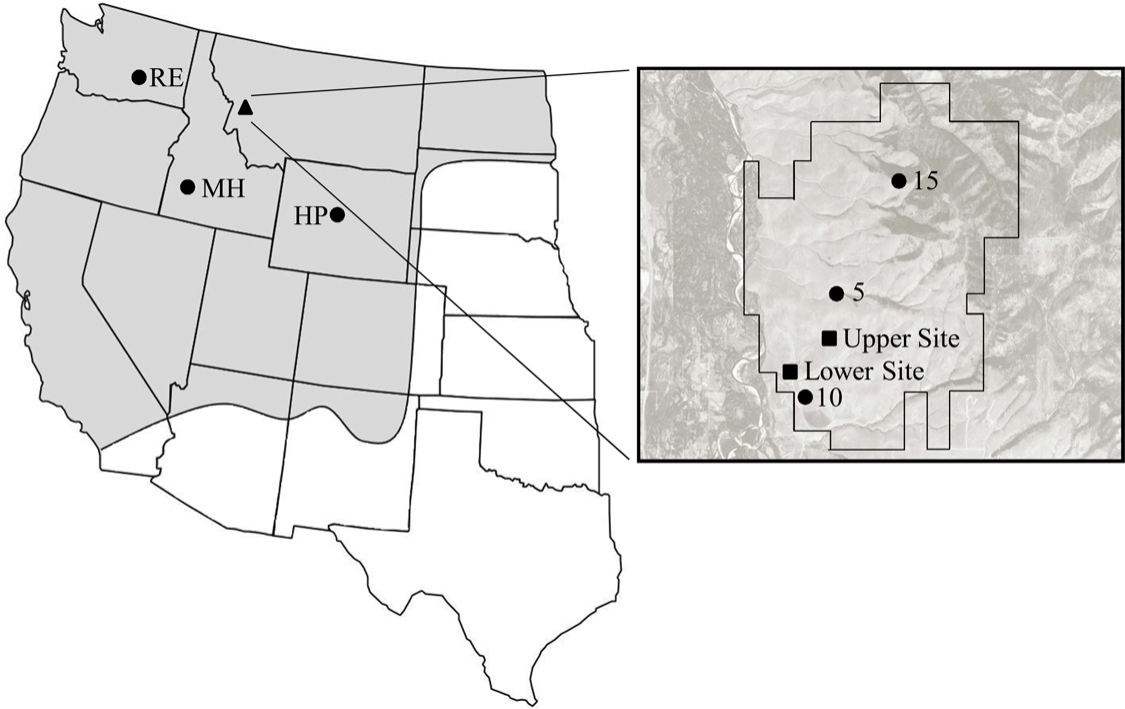
Origin of each *Poa secunda* accession. On the left is the distribution of *Poa secunda* in the contiguous western United States (shaded area; adapted from Stubbendieck, Hatch & Bryan 2011). The location of the MPG Ranch field site is indicated by the triangle. Circles indicate the origin locations of the cultivated accessions. To the right is an aerial image of MPG Ranch property lines. Circles mark the locations of the three wild seed source origins (MPG accessions 5, 10 and 15). The two experimental sites are indicated by squares.

We conducted this study at MPG Ranch, a conservation property consisting of 9,500 acres in western Montana (Fig. 1). The ranch is located on the west-facing slope of the Bitterroot Valley in the Northern Rocky Mountains. Prior to 2009, MPG Ranch had been used for agricultural production and livestock grazing for more than a century. Since that time, livestock have been removed and the majority of crop fields are out of production. Dominant native species present in MPG grasslands are *Pseudoroegneria spicata* (Pursh) Á. Löve, *Festuca idahoensis* Elmer, *Koeleria macrantha* (Ledeb.) Schultes, and *Poa secunda*. Invasive species are widespread and include *Centaurea stoebe* L., *Poa bulbosa* L. and *B*. *tectorum*.

Our experiment was conducted at two sites at MPG Ranch to evaluate the interactions of local environmental conditions and competition by exotic invasive plants with wild and cultivated accessions of *P*. *secunda*. The first (Lower) site was located at an elevation of 998 m and consisted of sandy loam soil having an average pH of 6.3. Prior to establishment of the experimental plots, the area had been used for soybean production. The second (Upper) site was located 1,023 m further up-slope, at an elevation of 1,076 m and consisted of loam soil with an average pH of 6.6. The Upper Site had previously been dominated by *Agropyron cristatum* (L.) Gaertn, (crested wheatgrass) with a *P*. *bulbosa* understory and used as pasture for cattle. Historical climate data for the area available from the PRISM Climate Group (2012) recorded an average annual precipitation of approximately 35 cm and a mean annual temperature of 7°C (range of -9–30°C). For the duration of this study, annual precipitation logged at the nearest MPG Ranch weather station was approximately 20 cm and the mean temperature was 9°C (-17–37°C).

### Study Species

*Poa secunda* is an important component of sagebrush steppe and temperate grassland vegetation in the western United States [28]. A native perennial bunchgrass, *P*. *secunda* serves as a primary source of forage for both wildlife and livestock at the beginning of the growing season [29]. *Poa secunda* occurs from Alaska through the Intermountain West and Great Plains states. Individuals of Sandberg bluegrass germinate early in the spring, and reproduce by tillering and seed maturation in early summer. Seed production is commonly the result of apomixis, but outcrossed pollination and subsequent self-fertilization contribute to distinct genetic lines [30].

Three U.S. Department of Agriculture selected varieties (hereafter, “cultivars”) were included in the study because of their recommended use in the study region and similar, early-maturing attributes within the *P*. *secunda* complex [26]. These cultivars included “High Plains Germplasm” (HP) with origins in Wyoming, “Mountain Home Germplasm” (MH) with origins in Idaho, and “Reliable” (RE) from Washington State (Table 1; Fig. 1). Cultivars were compared with three wild accessions (MPG-5, MPG-10, MPG-15) derived from sites that differed in elevation, aspect, and surrounding plant community. Both wild Sandberg bluegrass and cheatgrass seeds were collected at MPG Ranch during the summer of 2012 and subsequently stored at room temperature (15–25°C) until planting.

**Table 1:** Site characteristics at the location of origin for each *Poa secunda* wild and cultivated accession.

Accession	Approximate Location	Latitude, Longitude	Elevation (m)	Plant Community	Annual Temp. (°C)	Annual Precip. (cm)
MPG-5	Florence, MT	46° 41′ 35" N, 114° 1′ 5" W	1,144	*Bromus tectorum*, *Hesperostipa comata*, *Aristida purpurea*	7.0	35.0
MPG-10	Florence, MT	46° 40′ 18″ N, 114° 1′ 43″ W	1,026	*Pseudoroegneria spicata*, *Bromus tectorum*, *Poa bulbosa*	“	“
MPG-15	Florence, MT	46° 42′ 50″ N, 113° 59′ 49″ W	1,333	*Thinopyrum intermedium*, *Bromus tectorum*, *Pseudoroegneria spicata*	“	“
High Plains	Casper, WY, Granger, WY, Gillette, WY	42° 47′ 37″ N, 107° 35′ 54″ W	1,647	*Hesperostipa comata*, *Pascopyrum smithii*, *Bouteloua gracilis*	8.3	26.7
Mountain Home	Mountain Home, ID	42° 52′ 4″ N, 115° 34′ 57″ W	900	*Elymus elymoides*, *Bromus tectorum*, *Sisymbrium altissimum*	9.5	20.0
Reliable	Yakima, WY	46° 45′ 40″ N, 120° 11′ 29″ W	639	*Pseudoroegneria spicata*, *Artemisia tridentate wyomingensis*, *Achillea millefolium occidentalis*	11.6	21.0

### Experimental Design

We established and seeded our study sites in early fall 2012. The two sites were initially disked, chain harrowed, and raked to remove existing vegetation and litter to ensure a smooth and even seedbed. Fencing was installed around the perimeter of each experimental site to prevent herbivory by large ungulates.

The experiment was organized as a randomized complete block design with five paired-plot replicates at both study sites, resulting in ten plots per site (S1 Fig.). One plot of each pair was either weed-free or planted with *B*. *tectorum* (hereafter, *Bromus* or brome). Brome seeds were broadcast into experimental plots at a rate of 7,600 pure live seed m^-2^ [31, 32] and incorporated into the soil by raking before planting Sandberg bluegrass. Subsequently, all six *Poa secunda* (hereafter, *Poa*) accessions were replicated 50 times within each plot, and planted in random order at intervals of 30 cm to avoid intraspecific competition. To account for viability differences among seed sources, four seeds of the same accession were planted per replication to ensure high germination rates in the spring [33]. A color-coded toothpick was then placed in the center of the four planted seeds to aid in locating the plants and to identify which accession had been planted. When plants emerged in early spring, the four seedlings were thinned to one plant per replication. Due to drought conditions, plots were watered during the first week of May with a one-time application of approximately 30 ml applied to each plant via a sprinkler system.

Each plot had dimensions of 1.8 x 15 m and each pair of weed-free and competition plots was separated by 1 m. All plots contained a 45 cm wide buffer around the edge; buffer zones were cleared for weed-free plots, and planted with *Bromus* for competition plots to avoid edge effects. This experimental design resulted in a total of 300 *Poa* plants possible (50 replications for the 6 accessions) per plot.

### Sampling Method

Experimental plots were surveyed for germination during spring 2013 after seeds had overwintered. A survey of survival was subsequently conducted once every 3 weeks during the spring/summer growing season, resulting in four surveys for all experimental plots. A plot-wide survey of *Bromus* canopy cover was taken at its maximum production period in July to determine if levels of competition were even between study sites. *Bromus* cover was measured using Daubenmire’s six cover classes [34]. Sites were cleared of all non-experimental vegetation between surveys to ensure that relatively little competition occurred in the weed-free plots, and that competition was due to the presence of brome in the weed plots. At the same time, we noted the presence or absence of herbivory for each plant surveyed. At the end of the growing season, aboveground biomass for all *Poa* plants was clipped and dried in an oven at 60°C for three days. Dry weights for aboveground biomass were measured to 0.0001 g using an analytical balance (Mettler Toledo, Columbus, OH, U.S.A.).

### Statistical Analyses

Data for analyses comprised experimental plot means for each accession with respect to the proportion of survivors and average biomass. We calculated the proportion of survivors as the number of surviving seedlings divided by the original number of replications per plot (50 per accession). Average biomass was calculated for the number of survivors per accession per plot (*n* ≤ 50). Prior to analyses, survival data were arcsine square-root transformed and biomass data were log transformed to satisfy assumptions of statistical tests [35].

The repeated-measures data for survival were analyzed using a split-plot analysis of variance (ANOVA) where block was nested within site. Block was considered a random factor and accession, site, and competition treatment were analyzed as fixed factors. The repeated measures model incorporated compound symmetry covariance structure which resulted in the smallest Akaike’s Information Criterion [36].

A split-plot, mixed-model ANOVA (block nested within site) was used to calculate the significance of main and interaction effects for above-ground biomass of each cultivated and wild seed source between weed-free and competition plots. Block was treated as a random factor and accession, site, and competition treatment were analyzed as fixed factors. All calculations for survival and biomass data were made using the MIXED procedure in SAS version 9.3.1, using an alpha of 0.05 (SAS Institute Inc., Cary, NC, U.S.A.). The presence or absence of small mammal herbivory was added to the models as a covariate. Degrees of freedom were estimated using Satterwaite’s method [36] and paired comparisons were made using least-squares means. We tested assumptions of higher vigor among cultivars relative to wild populations with contrasts of the pooled seed sources (i.e. wild versus cultivar) and also examined the performance of individual cultivar and wild accessions.

As a last step, we assessed *Bromus* cover to ensure that the level of competition was consistent among all treatment plots in the study. The midpoint of the Daubenmire cover class range was averaged across each treatment plot. Average *Bromus* cover values were compared to determine if cover class was significantly different among competition plots.

## Results

A total of 2,627 seeds germinated and established, and 1,773 were monitored after thinning to one plant per replication. One of the wild seed sources (MPG-5) had near-zero rates of germination and as a result, this seed source was removed from analyses. Despite efforts to account for differences in seed viability, cultivated seeds had higher rates of seedling emergence when compared with the two wild seed sources (0.48 vs. 0.15, *P* < 0.001). This outcome may reflect differences in the viability or germination and establishment of seeds among accessions. Herbivory was noted during the final two surveys, and was likely caused by small rodents. Approximately 21% of experimental *Poa* plants were impacted by herbivory at the time of the third survey, and this number increased to 28% by the fourth and final survey. Average *Bromus* canopy cover for competition plots ranged from 46–69%. Plot cover differences were not significant and we included competition effects in analyses measured as the presence or absence of brome.

### Survival

Patterns of *Poa* survival were influenced by accession, competition, site, and survey, resulting in a significant four-way interaction in analysis of the full dataset (S1 Table). Due to the difficulty of testing and interpreting these results, we conducted exploratory data analysis guided by field observations of survival among survey periods. While the four-way interaction was explained to some extent by each of the factors included in the analysis, the survey period—particularly the fourth survey—was responsible for the significant effect. As a result, we report repeated measures analyses of survival data for the first three surveys separate from analysis of the final survey at the end of the growing season. Patterns of survival differed significantly among accessions in the senescent period at the end of the summer relative to data collected earlier in the growing season.

**Fig 2:**
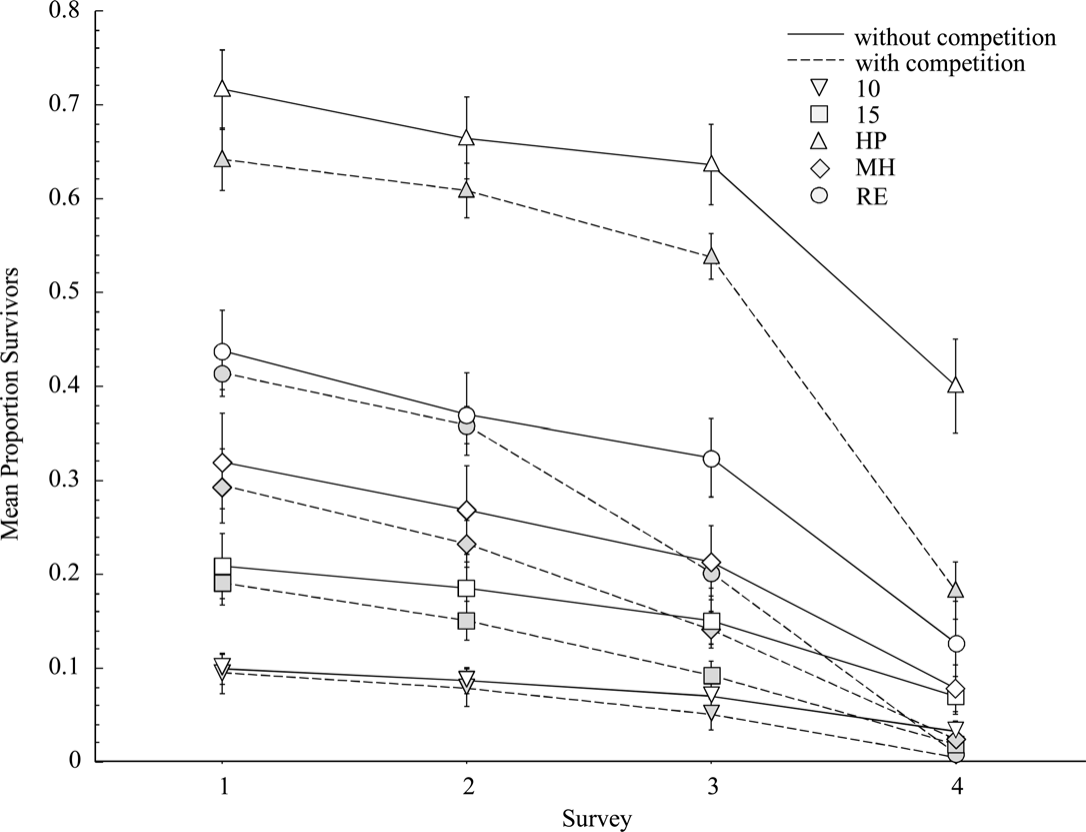
Average proportion survival per *Poa secunda* accession between non-competing and competing plants over time (± 1 SE).

**Fig 3:**
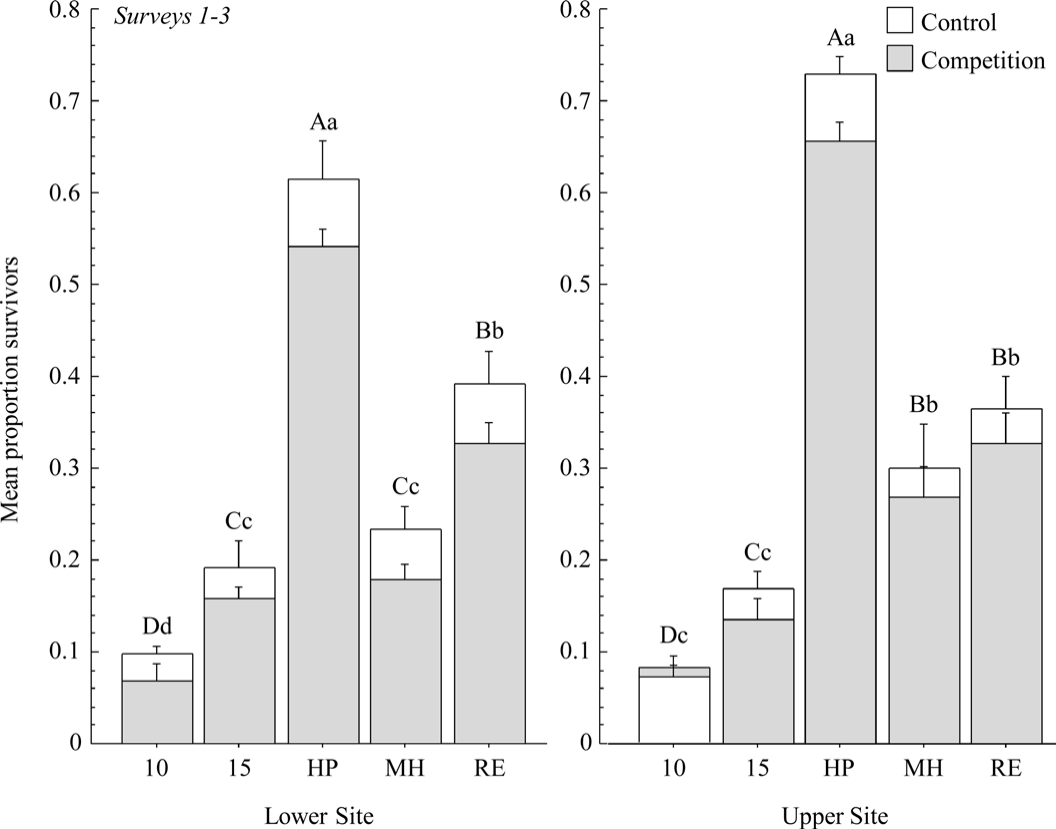
Average proportion survival per accession for surveys 1–3 between non-competing and competing plants (+ 1 SE). Different uppercase letters denote significant differences among non-competing accessions and lowercase letters denote differences among competing accessions. At the Upper Site, MPG-10 had a smaller mean proportion of survivors when grown alone, compared to when grown in competition.

Results of the repeated measures analysis for surveys 1–3 revealed significant differences in seedling survival for the interactive effects of accession, site, survey, and competition treatment (Table 2). Survival differed among each *Poa* accession (*P* < 0.001), and in all cases, survival declined over time (Fig. 2). We did not detect a significant effect of competition with brome for *Poa* survival early in the growing season (Fig. 3). Overall, cultivars were more likely to survive than wild accessions (*P* < 0.001) and survival of HP seedlings was greater than all other cultivated and wild accessions combined (*P* < 0.001).

**Table 2:** Repeated measures analysis of seed source, competition treatment, and planting location effects on *Poa secunda* proportion survival for surveys 1–3.

*Effect*	*Numerator DF*	*Denominator DF*	*F Value*	*Prob > F*
accession	4	64.7	227.22	<0.001
competition	1	16.1	1.33	0.265
site	1	16.8	0.07	0.800
survey	2	162	107.75	<0.001
accession × competition	4	63.7	0.18	0.948
accession × site	4	64.7	3.65	0.010
accession × survey	8	159	5.47	<0.001
competition × site	1	16.1	0.25	0.621
competition × survey	2	160	5.99	0.003
site × survey	2	162	17.22	<0.001
accession × competition × site	4	63.7	0.53	0.717
accession × competition × survey	8	159	1.73	0.096
accession × site × survey	8	159	1.36	0.220
competition × site × survey	2	160	0.73	0.484
accession × competition × site × survey	8	159	0.72	0.674
herbivory	1	167	6.93	0.009

**Fig 4:**
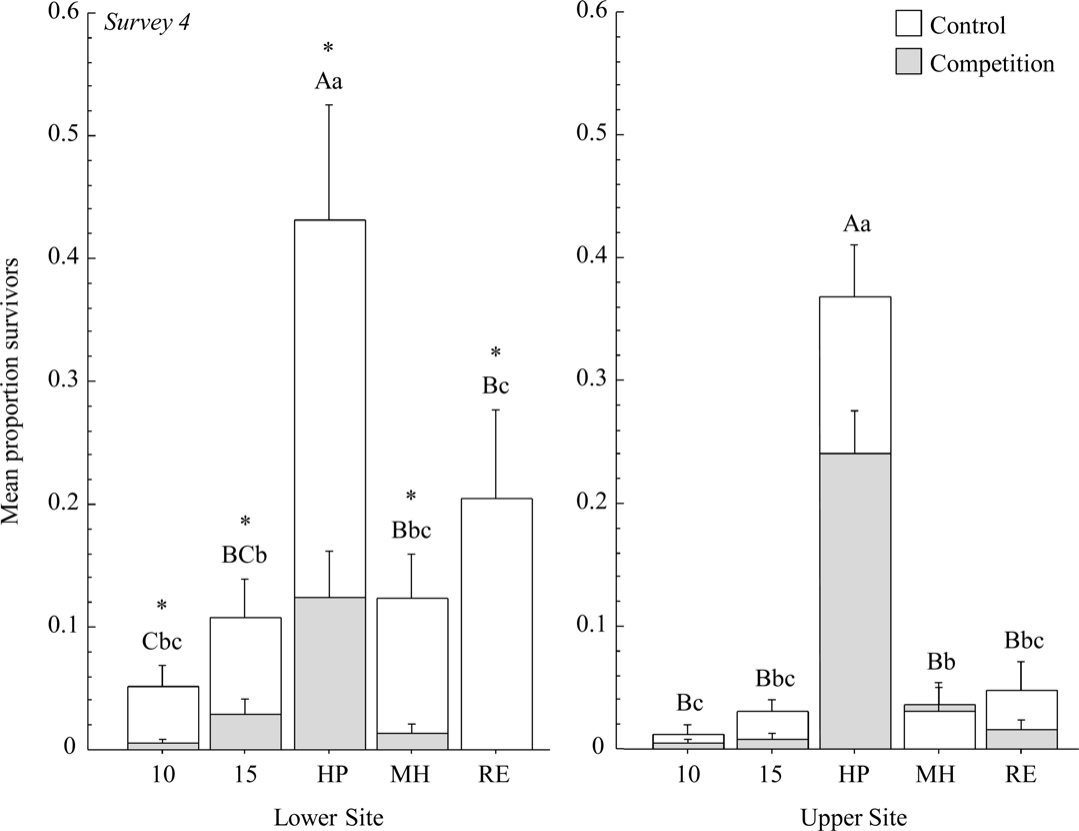
Average proportion survival per accession at survey 4 between non-competing and competing plants (+ 1 SE). Asterisks denote significances within accessions between competition treatments. Different uppercase letters denote significant differences among non-competing accessions and lowercase letters denote differences among competing accessions. At the Upper Site, Mountain Home had a smaller mean proportion of survivors when grown alone, compared to when grown in competition

At the end of the growing season, survival was significantly impacted by the interaction of *Poa* accession, competition treatment and differences between experimental sites (Table 3). Of note was the significant competition effect in weed plots (Fig. 4). Survival rates were lower for *Poa* seedlings grown with *Bromus* later in the season when plants were larger and competition for resources was greater. Survival rates were higher for all seed sources in weed-free plots at the Lower Site relative to competition plots. However, no differences were detected for any of the five seed sources at the Upper Site when survival in weed-free plots was compared with competition plots. Herbivory was greater at the Upper Site over the growing season, although this effect was not significant for survival at the final survey.

**Table 3:** ANOVA of *Poa secunda* proportion survival for survey 4.

Effect	Numerator DF	Denominator DF	F Value	Prob > F
accession	4	37.8	33	<0.001
competition	1	14.7	8.08	0.013
site	1	13.4	2.71	0.123
accession × competition	4	34.2	2.87	0.037
accession × site	4	35.4	1.16	0.343
competition × site	1	8.14	8.14	0.021
accession × competition × site	4	33.1	4.12	0.008
herbivory	1	66.7	1.6	0.210

Cultivars were more likely to survive than wild plants by the time of the last survey (*P* < 0.001), although that effect was solely the result of greater survival of the HP cultivar (cultivar vs. wild when HP removed, *P* = 0.207). Among accessions, the HP cultivar had the greatest number of survivors regardless of site or competition treatment (*P* < 0.001). We also noted that survival of MPG-15 seedlings did not differ significantly from MH and RE seedlings, suggesting that some wild populations may perform as well as some cultivated accessions. Only MPG-10 and MH had a similar proportion of survivors in competition and weed-free plots. However, the number of survivors was low for these two accessions and we were unable to determine if this effect was due to the ability of these accessions to tolerate *Bromus*, or the result of a lack of statistical power.

To sum, we noted significant differences in survival among the five accessions for both experimental sites, and survival differences between weed-free and competition plots for the Lower Site, at the fourth survey. The significant 3-way interaction at the final survey between site, accession and competition effects was explained by greater rates of herbivory at the Upper Site later in the growing season. The Upper Site was in a more remote location than the Lower Site, and it is likely that *Poa* seedlings were exposed to greater rates of herbivory in weeded plots, resulting in similar numbers of surviving seedlings between weed-free and competition plots over time.

### Biomass

*Poa* biomass was significantly influenced by accession and statistical interactions between accession, experimental site, and competition treatment (Table 4). Cultivars had greater biomass relative to wild plants (*P* = 0.025), although that effect was no longer significant when the HP cultivar was removed from the contrast (*P* = 0.482). HP produced the greatest average biomass compared to all other accessions (*P* = 0.0004). On average, the five accessions produced more biomass at the Lower Site than the Upper Site, but this difference was only significant for MPG-15. At the Lower Site, wild accessions performed as well as a subset of cultivars (MPG-15 performed as well as HP and MH, and MPG-10 was similar to MH and RE), while MPG-15 was similar to cultivars MH and RE at the Upper Site. Regardless of accession, competition with *Bromus* negatively affected *Poa* biomass at the Lower Site (*P* = 0.003). However, there was no effect of competition at the Upper Site (*P* = 0.237), and this was likely the result of high levels of herbivory that reduced biomass of *Poa* in weed-free plots by the final survey. Biomass did not differ between sites in competition plots.

**Table 4:** ANOVA of *Poa secunda* average biomass.

Effect	Numerator DF	Denominator DF	F Value	Prob > F
accession	4	16.8	6.61	0.002
competition	1	7.47	2.78	0.137
site	1	7.21	4.62	0.068
accession × competition	4	48	2.72	0.041
accession × site	4	49	4.72	0.003
competition × site	1	3.76	16.59	0.017
accession × competition × site	4	49	0.75	0.564
herbivory	1	50.4	36.05	<0.001

**Fig 5:**
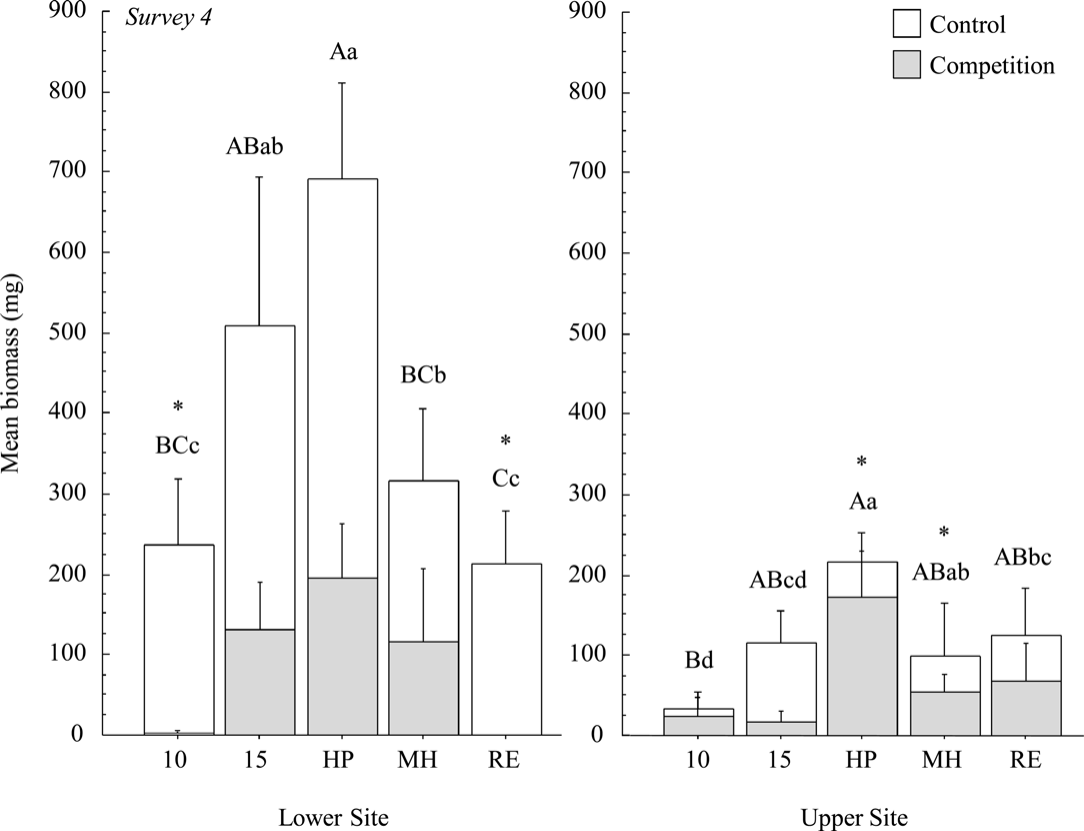
Average aboveground biomass of surviving plants at survey 4 per accession between non-competing and competing plants (+ 1 SE). Asterisks denote significance within accessions between competition treatments. Different uppercase letters denote significant differences among non-competing accessions, and lowercase letters denote differences among competing accessions.

Regardless of site, HP yielded the greatest biomass compared to all other accessions when in competition with *Bromus* (*P* < 0.001; Fig. 5). Cultivars, overall, produced significantly more biomass compared to wild plants when in competition (*P* = 0.007), but this effect was due to the performance of HP (cultivar vs. wild when HP removed, *P* = 0.232). Wild accessions did not differ from cultivars in weed-free plots (*P* = 0.372). Both wild accessions performed as well as at least two cultivars in weed-free plots (MPG-15 was similar to all three cultivars, while MPG-10 was similar to MH and RE), and they performed as well as one or more cultivars in competition plots (MPG-15 was similar to MH and RE, while MPG-10 matched RE). Three accessions (HP, MH and MPG-15) produced similar amounts of biomass between weed-free and competition plots, while two accessions (MPG-10 and RE) produced significantly lower biomass in competition plots relative to weed-free plots.

## Discussion

In the present study, we investigated seedling establishment and growth among local and cultivated genotypes of *Poa secunda* at sites impacted by cheatgrass invasion. We noted differences in performance among seed sources for measured variables in nearly all cases, suggesting that the source of seeds can have a significant impact on plant performance and, as a result, restoration outcomes [37]. Mean biomass and survival of cultivars was greater than wild plants on average, and these results reflect the increased vigor documented for cultivars relative to wild populations (e.g. [14, 38]). Differences in vigor were also apparent between local accessions, and MPG-15 had higher fitness and increased seed viability relative to MPG-10. Among the five accessions, performance of the High Plains cultivar was distinctive. High Plains seedlings maintained their overall performance advantage in both weed-free and competition plots. The High Plains cultivar was originally derived from high elevation sites with a semi-arid climate, which may have been an advantage when the plants were grown at exposed sites in this study [39]. Experimental plots imitated seedbeds for ecological restoration and likely represented relatively low soil moisture and extremes in temperature range, which negatively impact seedling establishment [40].

Competition effects in *Bromus* plots were only apparent late in the growing season and were significant at the final survey. This indicated a window of opportunity to minimize competition with weeds when restoration sites are cleared prior to seeding, and thereby improve establishment of desired species with continued management of exotics [41, 42]. Despite the overall advantage of cultivars, in each test, one or more wild accessions performed as well when compared to one or more cultivated accessions, particularly in competition plots. In addition, seedling performance also varied among cultivars and between the two wild seed sources. While High Plains seedlings had the greatest vigor among accessions in competition plots, the performance of Mountain Home seedlings exhibited the smallest decline in the presence of *Bromus* for all measured variables, indicating its potential for tolerance of invasive species. One of Mountain Home’s reported uses is in post-fire restoration of *Bromus* dominated landscapes [43], and the competitive ability of this cultivar warrants further investigation in the field.

Our study was a limited test of local adaptation because we were unable to reciprocally transplant seed sources, the resident plant community was removed prior to site establishment, and sites were weeded throughout the duration of the experiment [44]. Evidence for adaptive variation may only be apparent later in the life cycle and in competition with surrounding vegetation [45]. However, restoration commonly includes site preparation and weed control prior to seeding. Our results therefore provide key information for planting success of this cool season grass species early in restoration, and in the presence of one of North America’s most destructive invasive species. Successful establishment of native species is critical prior to invasion of exotic weeds at the restoration site [46]. A next step for evaluation of different seed sources of *P*. *secunda* would be to test the consequences of seed source over time once seedlings are established, and determine the replicability of these results when plants are grown in a matrix of the resident (native and non-native) plant community.

Environmental conditions such as soil, slope, precipitation, and elevation may have influenced differences in seedling performance between experimental sites. Low emergence rates across accessions could be explained by the drier than normal conditions that year. Early in the field season, survival of experimental plants was greater at the Upper Site where loam soils had more potential for moisture retention relative to sandy loam soil at the Lower Site [47]. During the second half of the growing season, plant performance at the Upper Site declined rapidly when compared to the Lower Site, and this was likely a result of differences in slope and rates of herbivory. Herbivory obscured variation among seed sources at the Upper Site, suggesting that grazing may reduce the advantage of dominant genotypes in the short term [48].

In summary, we found that these three cultivars had an overall advantage over local genotypes, supporting evidence of greater vigor among cultivated varieties of native species. This advantage, however, declined rapidly in the presence of *Bromus* and most accessions were not significantly different for growth and survival in competition treatments. Only the HP cultivar had an advantage, and the rapid decline in its performance in the presence of *Bromus* indicated this advantage may decline over time and requires further investigation. As a result, cultivated varieties as a group did not meet our expectations for greater establishment and persistence relative to local plants in the presence of invasive, exotic species. Other control measures would be needed to reduce competition with invasive plants, and promote the early establishment of desirable, native vegetation (e.g. herbicide; [49]). Additionally, numerous studies suggest that the differences in vigor between cultivated and non-cultivated varieties can impact community-level interactions and population dynamics [7, 8, 50] (but see [51]). Non-cultivated, wild populations therefore remain the best choice to maintain population genetic diversity and avoid risks of large-scale introductions of non-local genotypes [1]. Measures should be taken to ensure high viability of seed stock before planting to improve establishment.

In cases where sites are heavily impacted by invasive species and other control measures are not feasible or are ineffective, our results suggest cultivated varieties may provide some advantage for establishment of natives. However, the ability to discern among cultivars is difficult unless data are available for competitive dominance and overall vigor (such as in the case of High Plains). If no data are available, the use of multiple cultivar accessions might increase the odds of including highly competitive genotypes in the restoration seed mix. This strategy, however, is not without risks and would be most useful in highly invaded settings. In contrast, if restoration sites are contiguous with undisturbed plant communities and/or exotic species are not predominant, we recommend the use of a composite seed mix representing appropriate local or regional variation either via wild collections or limited field production of region-specific varieties to avoid directional selection and altered patterns of genetic variation. This approach would maintain intraspecific patterns of genetic diversity, increase the odds of including vigorous wild genotypes, and may improve plant community diversity as well as resistance to biological invasion [6–8, 52].

## Supporting Information

S1 Fig Experimental design of each study site. Each site contained five blocks (Block 1–5), split (Plot A or B) between one weed-free plot (white border) and one paired cheatgrass plot (shaded border). Every plot contained 50 replicates of all six *Poa secunda* accessions, placed randomly within each plot (each small square cell within a plot corresponded to one *Poa* replication).
(TIFF)

S1 Table Repeated measures analysis of seed source, competition treatment, and planting location effects on *Poa secunda* proportion survival for surveys 1–4. (DOCX)
