# Supplementary figures and images for: Consequences of Seed Origin and Biological Invasion for Early Establishment in Restoration of a North American Grass Species
Source: PLoS One. 2015 Mar 5;10(3):e0119889. doi: 10.1371/journal.pone.0119889 (PMC4351099; doi:10.1371/journal.pone.0119889)

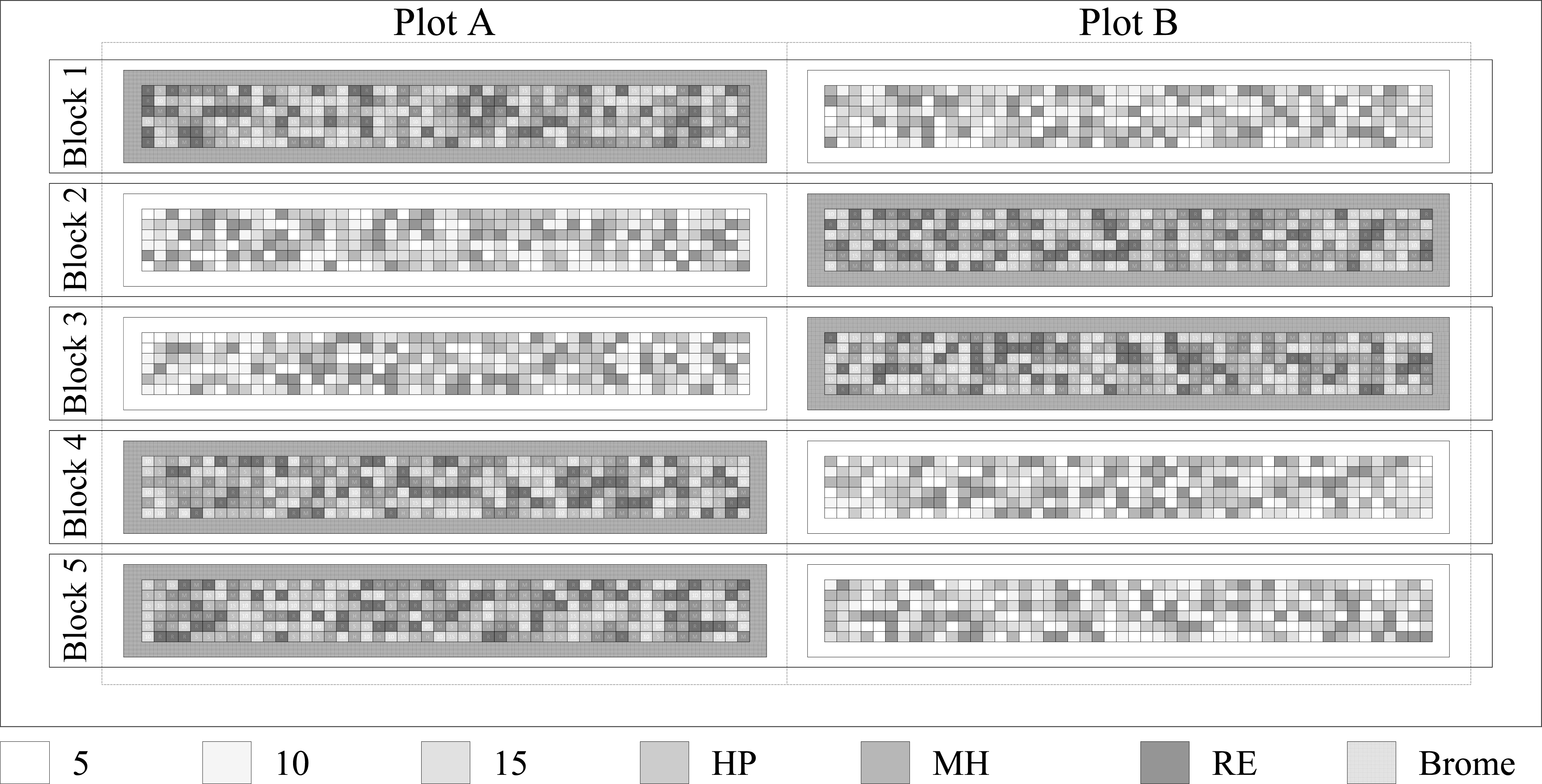

Supplement: S1 Fig — Each site contained five blocks (Block 1–5), split (Plot A or B) between one weed-free plot (white border) and one paired cheatgrass plot (shaded border). Every plot contained 50 replicates of all six Poa secunda accessions, placed randomly within each plot (each small square cell within a plot corresponded to one Poa replication). (TIFF) [file pone.0119889.s001.tiff]
