# Supplementary material for: Consequences of Seed Origin and Biological Invasion for Early Establishment in Restoration of a North American Grass Species
Source: PLoS One. 2015 Mar 5;10(3):e0119889. doi: 10.1371/journal.pone.0119889 (PMC4351099; doi:10.1371/journal.pone.0119889)
Supplement: S1 Table — (DOCX) [file pone.0119889.s002.docx]

**Supporting Information**

**S1 Table.** Repeated measures analysis of seed source, competition treatment, and planting location effects on *Poa secunda* proportion survival for surveys 1-4.

| **Effect** | **Numerator DF** | **Denominator DF** | **F Value** | **Prob > F** |
| --- | --- | --- | --- | --- |
| accession | 4 | 34.7 | 210.42 | <0.001 |
| competition | 1 | 8.2 | 3.38 | 0.102 |
| site | 1 | 8.84 | 0.02 | 0.888 |
| survey | 3 | 248 | 276.46 | <0.001 |
| accession × competition | 4 | 31.7 | 0.99 | 0.428 |
| accession × site | 4 | 33.7 | 4.11 | 0.008 |
| accession × survey | 12 | 239 | 16.93 | <0.001 |
| competition × site | 1 | 8.04 | 1.12 | 0.320 |
| competition × survey | 3 | 244 | 11.69 | <0.001 |
| site × survey | 3 | 247 | 13.82 | <0.001 |
| accession × competition × site | 4 | 31.3 | 0.82 | 0.522 |
| accession × competition × survey | 12 | 238 | 1.66 | 0.077 |
| accession × site × survey | 12 | 238 | 0.91 | 0.534 |
| competition × site × survey | 3 | 240 | 18.9 | <0.001 |
| accession × competition × site × survey | 12 | 237 | 1.87 | 0.039 |
| herbivory | 1 | 278 | 2.33 | 0.128 |
